# Failure of ALL recognition by CAR T cells: a review of CD 19-negative relapses after anti-CD 19 CAR-T treatment in B-ALL

**DOI:** 10.3389/fimmu.2023.1165870

**Published:** 2023-04-14

**Authors:** Clara Aparicio-Pérez, MDolores Carmona, Karim Benabdellah, Concha Herrera

**Affiliations:** ^1^ Department of Hematology, Reina Sofía University Hospital, Cordoba, Spain; ^2^ Maimonides Institute of Biomedical Research in Cordoba (IMIBIC), Cell Therapy, Cordoba, Spain; ^3^ Red de Investigación Cooperativa Orientada a Resultados en Salud-Terapias Avanzadas (RICORS-TERAV), Carlos III Health Center (ISCIII), Madrid, Spain; ^4^ Department of Genomic Medicine, Pfizer-University of Granada-Andalusian Regional Government Centre for Genomics and Oncological Research (GENYO), Granada, Spain; ^5^ Medicine Department, University of Cordoba, Cordoba, Spain

**Keywords:** CART cells, B-ALL, CD-19-negative relapse, leukemia evasion, overcome strategies

## Abstract

The use of chimeric antigen receptor (CAR) T lymphocytes in the treatment of refractory or relapsed (R/R) B cell acute lymphoblastic leukemia (B-ALL) has meant a radical change in the prognosis of these patients, whose chances of survival with conventional treatment are very low. The current probability of event-free survival by R/R B-ALL patients treated using anti-CD 19 CART cell therapy is as high as 50-60% at 1.5 years, which is a very important advance for this group of very ill patients. Although most patients (70 to 94%) achieve complete remission (CR), the main problem continues to be relapse of the disease. Most relapses, both in clinical trials and real-world evidence, are due to failure of CAR-T cell expansion or limited CAR-T persistence. However, despite the adequate functioning of infused CART lymphocytes, the tumor cells of an important group of patients manage to evade CAR-T attack, resulting in a CD 19-negative relapse. Several mechanisms have been described that may be able to produce the escape of leukemic cells, such as acquired mutations and alternative splicing of the CD19 antigen, CD19 epitope loss or masking, leukemia lineage switching, and trogocytosis. In the present review, we comprehensively analyze the leukemic cell escape mechanisms, the incidence of CD19-negative relapse reported in clinical trials and real-world evidence (outside clinical trials), and provide an update on the main lines of current research into the prevention of leukemia evasion.

## 1 Introduction

Acute lymphoblastic leukemia (ALL) is the most common hematological cancer in children and young adults (1, 2) and, despite progression-free survival (PFS), around 80-90% of relapsed/refractory (R/R) pediatric cases with current chemotherapy regimens have a poor prognosis and a high mortality rate with conventional treatments (3).Over the last decade, immunotherapy with chimeric antigen receptor T (CAR-T) cell immunotherapy has been introduced into the therapeutic arsenal for these patients with impressive results compared with previous standard of care based on current chemotherapy regimens. The documented rate of R/R ALL-B prior to CAR-T cells complete remission (CR) rate range from 10-70%. However, with this immunotherapy CR rates range from 70-94%), most of whom present negative minimal residual disease (MRD), which was unthinkable with previously available treatments for this group of patients. However, around 35-55% of responding patients eventually relapse (4–10).

Most CAR-T cell products for B-cell neoplasms use autologous T cells engineered to express a CD19-specific CAR with 4IBB/CD3ζ or CD28/CD3ζ signaling, which is transduced using lentiviral or retroviral vector technology (11).Currently, there are two Food and Drug Administration (FDA) and European Medicines Agency (EMA)-approved CAR-T products for R/R B-ALL patients: tisagenlecleucel (previously CTL109 based on CAR construct anti-CD19scFv or FMC63 with 4IBB/CD3ζ signaling) (Kymriah^©^) is indicated for the treatment of children and young adults up to 25 years of age, and brexucabtagene autoleucel (Tecartus^©^) for adults. As most of the clinical data currently available correspond to tisagenlecleucel, which was approved for clinical use in 2017 in the USA and in 2018 in Europe, in addition to the clinical trials data prior to its approval (5, 9), an important data set pertaining to its use in the real world now exists (12–18). Given that Tecartus^©^ was approved in 2021, we only have the clinical trials data that led to its approval (19–22). We also have clinical data from R/R B-ALL patients treated in clinical trials with other CAR-T cells not approved for clinical use, with anti-CD19 specificity and anti-B markers such as CD22, co-stimulated by 4-1BB or CD28.

In all series and with all the CAR-T cell products used, significant CR rates are achieved, although relapse invariably occurs in 35-55% of patients who achieve remission, so that relapse is the main cause of failure of this otherwise very successful therapy. After treatment with CD19 CART cells, relapse of both the original CD19-positive leukemia and unexpected CD19-negative relapse have been observed, which is quite exceptional outside the context of immunotherapy. It is now clear that the causes of these two types of relapses are completely different. While CD19-positive relapses (70-80% of patients) are produced by mechanisms inherent to CAR T cells, which are unable to expand or persist in the circulation, CD19-negative relapses (20-30% of patients) are produced by evasion mechanisms inherent to leukemia and occur in the presence of perfectly functioning CART cells from which it manages to escape (23).

CD19-negative relapses carry a worse prognosis and create a major problem for potentially suitable subsequent treatments (15). The aim of this review is to summarize the biological mechanisms described as causing the loss of CD19 antigen and subsequent leukemia escape after anti-CD19 CAR T therapy, as well as the available clinical data on CD19-negative ALL relapses. And most importantly, we will provide an overview of current strategies in CART cell design, as well as of clinical approaches to therapy to avoid this very serious problem.

## 2 CD19-negative relapse mechanisms

CD19, a membrane protein belonging to the immunoglobulin super family, is expressed in the B-lymphoid lineage from its early mature stages and is lost during maturation to plasma cells. It is a key molecule in the maturation and differentiation, as well as in the regulation, of B lymphocytes in peripheral blood (24, 25). In ALL, it is expressed in almost 100% of newly diagnosed cases, making CD19 an ideal target for CAR-T cell therapy due to both its almost universal expression in ALL and its restriction to normal B lymphocytes which are “dispensable” cells.

**Figure 1:**
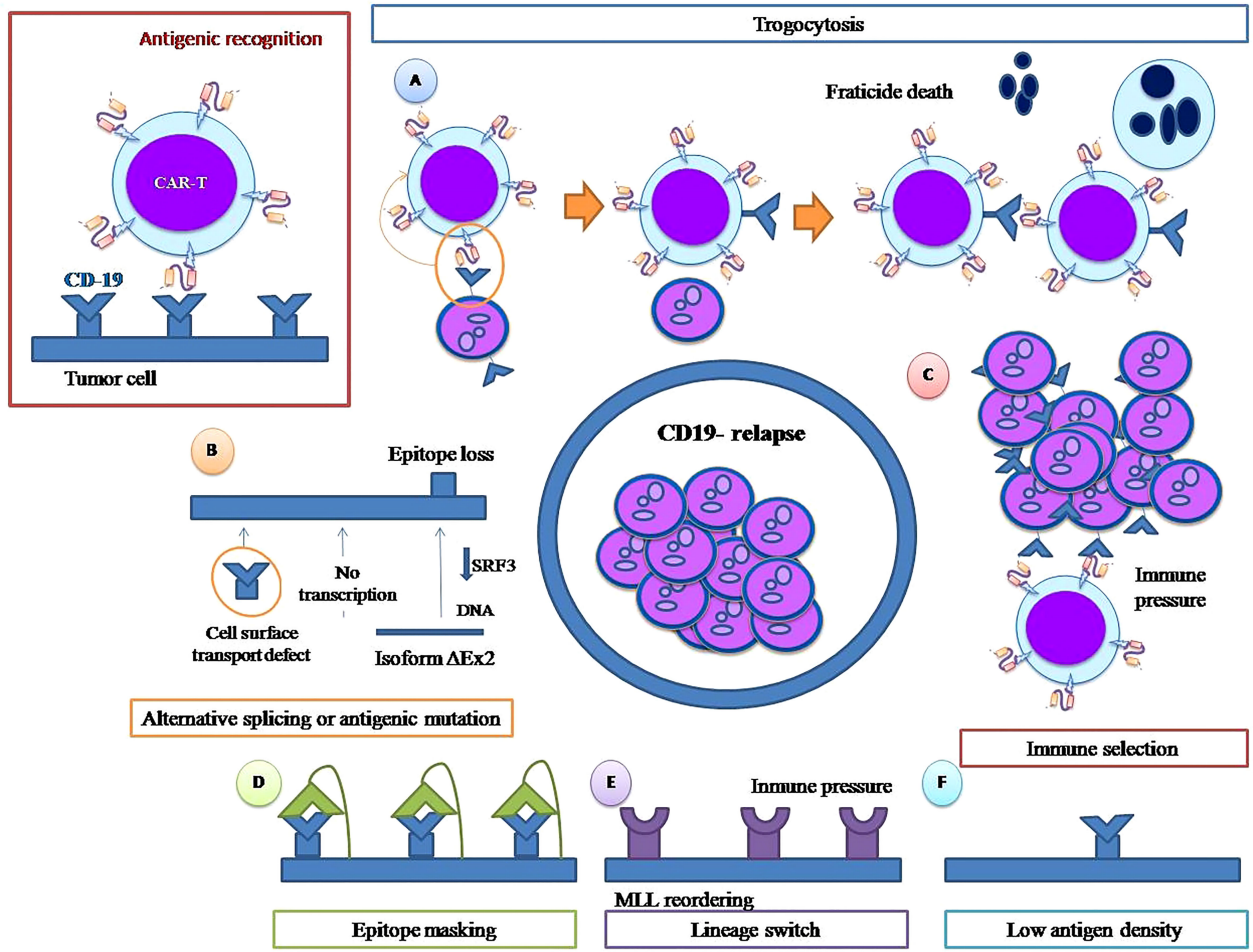
CD 19 negative relapse mechanisms. Trogocytosis and fratricide **(A)**. Alternative splicing or antigenic mutation **(B)** Immune pressure **(C)**. Epitope masking**(D)** Lineage switch**(E)**. Low antigen density **(F)**.

As CD19 is essential for the development of the B lineage (26), its loss is a striking form of leukemia relapse. Although CD19 is important for B-cell biology because of its role in B-cell receptor signaling, several biological and molecular mechanisms have been described as responsible for the appearance of CD19-negative ALL blasts after CART therapy which are ultimately responsible for relapse (**Figure 1**).

### 2.1 Immune pressure selection

In general terms, immune pressure selection refers to when immune-targeted therapy selects tumor cells, transforming pre-existing non-targeted tumor clones into dominant ones, leading to cancer relapse. It is that simple, without the intervention of any other molecular mechanism, and necessarily presupposes the existence of previous leukemic clones not carrying the target antigen.

In the first CD19-negative relapse after CTL019 infusion described in the literature by Grupp et al. in 2013 (4), the absence of the original dominant CD45dim+CD34+CD19dim+ clone was demonstrated, consistent with potent selective anti-leukemic pressure from CTL019. However, deep sequencing of DNA isolated from bone marrow cells obtained at the time of relapse revealed that the CD45+dimCD34+CD19neg cells and the initial dominant CD45dim+CD34+CD19dim+ clone shared the same IGH sequence and were therefore clonally related.

This has been demonstrated by a single-cell RNA sequencing (scRNA-seq) study of an ALL-B patient who relapsed with CD19-negative B cell ALL after anti-CD19 CAR T-cell therapy, in which it was shown that CD19-negative leukemic cells were present prior to CAR T-cell therapy, which would have caused the CD19-negative relapse by simple immune pressure (27).

Fischer et al. have analyzed bone marrow and peripheral blood samples from 14 children and 6 adults with CD19+ B-ALL, as well as normal non-leukemic controls. They found that the expression of a CD19 mRNA isoform lacked the CART-19 epitope in all the samples studied both in leukemic blasts and normal bone marrow from controls. This finding demonstrates that some of the CD19 isoforms that contribute to CART-19 escape are already present at diagnosis and could become the dominant clones during CART-19 therapy (28). In this line of evidence, CD34+CD19-CD22+ leukemic cells have recently been found at diagnosis and relapse in the bone marrow of 70% of B-ALL patients. This frequency doubles in CR patients after CAR CD19 T-cell therapy due to immune clearance of CD19-positive blasts. The median of CD34+CD19-CD22+ cells prior to treatment in this series was threefold higher in B-ALL relapsed patients after CD19-directed immunotherapy at a median follow-up of 24 months (29).

In all the above-mentioned cases, it seems clear that immune pressure simply selects preexisting CD19-negative clones directly related to the initially dominant CD19-positive clones. However, immune pressure also induces active mechanisms that leukemic cells use to avoid targeting and elimination by CD19 CAR-T cells, which is considered a cross-cutting determinant underlying all the mechanisms of CD19-negative relapse described.

### 2.2 Alternative splicing and acquired mutations

The CD19 gene contains 13 exons: exons 1 to 4 encode the extracellular domains, while exons 5 to 13 encode the transmembrane domains of the CD19 protein (30). Genetic mechanisms that can cause the loss of the CD19 antigen have been described both at the transcriptional level (i.e. alternative splicing) and at the genomic level as acquired mutations.

Alternative splicing (AS) is a post-transcriptional process by which introns are cleaved from the primary mRNA transcript, and exons are joined in different combinations to generate diverse mature mRNA transcripts (31). These mRNAs can be translated to produce different proteins with structures and functions distinct from a single gene (32). In tumors, AS favors targeted therapy evasion and is one of the mechanisms that has been involved in loss or altered expression of CD19 leading to ALL escape from CAR-T anti-CD19 therapy (28).

Distributed frameshift mutations can prevent CD19 protein expression but can also seriously affect the development and functions of leukemic blasts for which CD19 signaling is required (33). However, AS can generate CD19 isoforms that modify the recognition epitope and/or reduce expression on the cell surface without the complete loss of the protein, thus maintaining some of its functions (23).

To study the mechanisms and consequences of CD19 loss *in vivo*, Sotillo et al. reanalyzed samples from CD19-positive pre-CAR-T-19 leukemia and relapsed CD19-negative leukemia obtained from the same patients. In patient CHOP101R, they found two frameshift mutations in exons 2 and 4 and, in patient CHOP133, a hemizygous loss of chromosome 16, as well as a frameshift mutation in exon 2, that generated nonsense mRNA (34). However, samples from other patients did not present mutations that could explain the loss of CD19 expression, which led the authors to look for changes at the transcriptional level. They found an isoform of exon 2 (Δex2), mainly cytosolic, which hides the recognition epitope from CAR T cells preventing cell attack. This isoform partially retains CD19 functionality and is even more stable than the complete CD19 protein which supposes an important advantage for leukemic cells in relapsed ALL (34).

Orlando et al. (6) analyzed samples from 12 patients with CD19-negative leukemic relapse after CAR T therapy and observed mutations in the CD19 domain in all of them. Each patient presented at least one distinct insertion or deletion in the reading frame of exons 2-5, some of them with single nucleotide variants, and all mutations identified by DNA-seq were confirmed by RNA-seq. Allelic frequencies of the mutations strongly correlated with the percentage of CD19-negative cells, as determined by flow cytometry, suggesting that homozygous or biallelic frameshift mutations in CD19 were the main cause of CD19 loss and acquired resistance to CTL019. AS determined by the rMATS algorithm occurred at extremely low frequencies (0–2.7%) and accounted for an insignificant fraction of the tumor cells. Moreover, similarly infrequent alternatively spliced mRNA isoforms were found for other genes, suggesting that AS is coincidental and not the cause of CD19-negative relapses (6).

### 2.3 CD19 epitope masking

Ruella et al. reported a case of CD19-negative relapse in which the leukemic blasts presented CAR cells on their surface. After lymphodepletion, CAR-T cells (CTL019) were infused into the patient who achieved CR on day 28 after infusion. However, routine monitoring by PCR of peripheral blood for CAR-specific sequences identified the appearance of a second phase of CAR construct expansion starting at day +252, which did not correlate with CAR+ T-cell re-expansion measured by flow cytometry. At day +261, the patient relapsed with the presence of more than 90% bone marrow CD10+, CD19- blasts and circulating blasts. However, despite CD19 negativity determined by flow cytometry, leukemic cells maintain abundant CD19 transcripts, as well as cytoplasmic CD19 protein, as determined by immunohistochemical staining. Confocal microscopy demonstrated colocalization of CAR19 and CD19 on the cell surface of the relapsed leukemia. The CAR transgene, which had been accidentally transduced into one leukemic cell during the manufacturing process, binds in the cis region to the CD19 epitope on the surface of leukemic cells, thus masking the epitope from detection by standard flow cytometry and by neighboring CAR T cells. Although leukemic cells persist in expressing CD 19 on their membrane, the transcription of CAR in their genome and its expression on the cell membrane neutralizes the expression of the CD 19 antigen and prevents it from being recognized both by the cellular response and by flow cytometry and immunohistochemistry techniques. Therefore, the presence of blasts “masked” with the transduced CAR transgene allowed the leukemic cells to escape cellular attack, resulting in apparently CD19-negative relapse (35).

All current CAR T-cell production processes include the selection of T lymphocytes prior to transduction to avoid this type of accident.

Anti CD19 immune-mediated therapies can also induce epitope masking. Fitzgerald et al. reported two cases of CD19 epitope masking in patients with aggressive mature B-cell lymphomas treated with Tafasitamab, an anti-CD19 antibody, in combination with lenalidomide. The loss of detectable cell surface CD19 was transient, which forced the delay of CAR T infusion in order to avoid interference with the efficacy of therapy (36).

### 2.4 Lineage switch

Another mechanism associated with CD19-negative relapse after CAR-T therapy is lineage switching (**Figure 1**), which is much more than the simple loss of the CD19 antigen, constituting a complex change from a B lymphoid phenotype to a myeloid phenotype. Jacoby et al. demonstrated that persistent CD19 CAR-T-cell immune pressure can induce lineage switch as a mechanism of CAR resistance (37). In their murine model, they found that long-term immune pressure exerted by the persistence of circulating anti-CD 19 CAR T cells can trigger B-cell lineage reprogramming, leading to late relapse in the form of Acute Myeloid Leukemia (AML). In this murine model, early CD19-negative relapse did not imply a lineage switch; CD19 was lost by an alternative splicing mechanism, and ALL cells maintained the rest of their pre-CAR exposure phenotype. However, late CD19-negative relapses showed a change in the transcriptional profile, with loss of transcription factors, such as Pax 5 and Ebf1, that regulate B-cell development and gain of significant myeloid transcription factors such as Cebp, Batf2 and Irf2, consistent with a genuine lineage switch. After lineage change, tumor cells lost B markers and acquired markers from other lineages such as CD11b, Gr 1 and cKIT that remained stable during follow-up. These studies clearly demonstrate that, under immune pressure maintained over time, ALL cells can undergo a lineage switch that requires considerable leukemia plasticity. This plasticity is genetically conditioned, as it does not occur equally in all cell lines; in fact, in the experiments carried out by Jacoby et al, under identical circumstances of prolonged immune pressure, ALL cells of the E2a:PBX1 line undergo lineage switch but not those of the Eμ-RE line. Similarly, CRISPR/cas 9 deletion of the lymphoid transcription factors Pax 5 and Ebf1 produces a lineage shift toward myeloid lineage in the cells of E2a:PBX1 ALL but not in the Eμ-RE cell line (37).

Besides the CAR T-driven lineage switch demonstration in this elegant murine model, clinical data are available that support this mechanism as a cause of CD19-negative relapse after CAR T cell therapy. Myeloid transformation of B-ALL had already been described in the pre-CAR T era in patients receiving chemotherapy, particularly in those with rearrangements of the mixed lineage leukemia (MLL1, KMT2A) gene at 11q23 (38, 39). KMT2A rearrangements occur as the initial or unique genetic lesion in >75% of infants with B-ALL and rarely in children and adults, producing leukemia with mixed myeloid and B-lymphoid characteristics and a uniformly worse prognosis (40). Lineage switch has been described after CART therapy with or without KMT-2A rearrangement (18, 41). Lamble et al. reported on 420 R/R B-ALL patients treated with CD19 CAR Tells,of whom 166 relapsed, with 12 (7.2%) showing lineage switch at relapse. KMT2Ar, the predominant cytogenetic abnormality seen in patients with lineage switching, was present in nine out of 12 (75%) patients compared to 20 out of408 (7.1%) patients who did not present lineage switching. The presence of a prior KMT2A rearrangement was the only pre-infusion risk factor associated with lineage switching in this series (18).

Garner et al. (42) treated seven KMT2Ar B-ALL patients with CD 19 CAR T cells. All patients achieved CR, but, within one month of CAR-T-cell infusion, two of the patients relapsed, as AML myeloid lineage blasts at relapse could arise due to cellular reprogramming, dedifferentiation of B lymphocytes, differentiation of non-target pre-B lymphocytes, or clonal replacement due to the presence of AML clones prior to cell therapy. The leukemic blasts in the two cases of relapse changed from a lymphoid to a myeloid phenotype, apparently by two distinct mechanisms: the first case involved an adult in which the myeloid blasts maintained the IGH rearrangement, suggesting a contribution of cellular reprogramming or dedifferentiation of previously committed B-lymphoid blasts, whereas the absence of the IGH rearrangement in the myeloid blasts of the second case is consistent with myeloid differentiation of a previously uncommitted B-lymphoid precursor. Taken together, these findings lead to the conclusion that, rather than clonal selection of preexisting myeloid neoplastic cells, myeloid blasts are the result of a conversion of lymphoid blasts caused by the interaction between the microenvironment and the presence of predisposing genetic rearrangements. Interestingly, neither the two patients who relapsed nor any of the non-relapse patients had severe cytokine release syndrome (CRS) associated with IL-6 release. IL-6 is a critical factor for the myeloid differentiation of a t (4, 10) MLLB- ALL line *in vitro*. Therefore, high serum IL6 levels during CRS could have led to myeloid differentiation of lymphoid clones (42).

In addition, data are available from 15 infants treated with CAR-T CD19 or CD19-CD22 in two clinical trials, PLAT-02 (NCT02028455) and PLAT-05 (NCT03330691), and from 14 more in the real world. Among the 15 patients included in the clinical trials, one relapsed with B-ALL and one with AML. The incidence of lineage switch among this infant ALL group was 1/15 (6.7%) (43). Of the 14 infants treated in the real world, the majority (86%) had KMT2Ar. Four patients, three of them with KMT2Ar B-ALL, showed conversion to AML either during primary response to CAR or during relapse (16).

### 2.5 Low surface antigen density

Unlike natural T cell receptor (TCR)-mediated signaling, CAR-T cells require a high density of the target antigen to fully activate and exert *in vivo* activity. These differences in antigen density requirements may emerge from the dramatic differences in structure between natural TCRs and CARs. TCRs possess multiple co-stimulation domains (gamma, delta, epsilon, zeta), whereas CAR cells incorporate the ζ chain as a single signaling element. During antigenic recognition, the TCR creates a very complete immunological synapse that includes co-receptors, allowing antigenic recognition with very low antigenic density. Current studies show that the CAR-generated immunological synapse is less organized (44). Antigenic binding to the receptor also differs. Natural TCRs are low-affinity receptors whereas single-chain fragment variables (scFvs) incorporated into CARs present high affinity. Thus, these differences may affect the quality of the response as a function of the density of antigen present, although this issue has not been fully characterized (45).

Majzneret al. (46) tested the CD19–4-1BBζ CAR construct in an *in vitro* assay against ALL cell line NALM6 clones expressing different amounts of CD19 on their surface. CD19–4-1BBζ CAR T cells demonstrated reduced killing capacity, as well as reduced proliferation and cytokine production, in response to clones expressing low levels of CD19 compared to those expressing high levels. In contrast, the CD19–28ζ construct was able to kill and proliferate in response to tumor cells expressing low levels of CD19. At all antigen densities tested, CD19-28ζ CAR T cells produced more IL-2 than CD19-4-1BBζ CAR T cells, and, at low antigen densities, only CD19-CD28ζ produced measurable amounts of IL-2.

Therefore, it is possible to assume, at least theoretically, that, in the absence of genetic mechanisms leading to a total loss of the CD 19 antigen, a simple down-modulation that reduces the number of CD 19 molecules on the membrane surface could lead to the escape from the leukemia of the CAR CD19–4-1BBζ employed in tisagenlecleucel.

The down-regulation of CD19 mediated by anti-CD19 CAR T cells has not been described in ALL cells, but it has been demonstrated in normal B lymphocytes and chronic lymphocytic leukemia (CLL) cells. Normal B lymphocytes down-regulate CD19 expression in the presence of anti-CD19 CAR T cells both *in vitro* and *in vivo* in a murine model. This phenomenon is reversible, so that CD19 expression is restored when cells are removed from co-culture with CAR T cells or when they are transferred from a CAR-T treated animal to an untreated animal (47). In CLL, a recent study found that down-regulation of CD19 expression, both in cell lines and primary cells, is caused by promoter DNA hypermethylation and is partially reversible by treatment with a demethylating agent (48).

In a clinical setting, relapses after CD22-targeted CAR T cell treatment have been associated with diminished CD22 site density without detectable CD22 mutations or changes in CD22 mRNA levels (49). However, the group of University of Pennsylvania (UPENN)reported their results in 166 cases of ALL treated with tisagenlecleucel, in which CD19 antigenic density was tested prior to CAR T infusion and, surprisingly, no significant differences in CAR-T cell efficacy were observed in CD19-dim B-ALL cases as compared to CD19-normal or -bright B-ALL cases (50). In cocultures conducted with primary ALL cells from these patients, CAR T cells recognized and lysed cells with very low levels of CD19 expression *in vitro*. The presence of CD19 dim or CD19-negative events, as measured by flow cytometry, did not predict no response or recurrence after CAR-T cell therapy in this series. According to these data, the only cases of leukemia that might not respond to tisagenlecleucel are those that are predominantly or completely negative for CD19.

Therefore, the mechanism of CD19 antigen down-regulation after CAR T therapy as the unique cause of leukemic relapse is not fully established: on the one hand, the induction by CAR T cells of CD19 down-regulation in ALL has not been described *in vitro* and, on the other hand, UPENN data seem to demonstrate that tisagenlecleucel is equally effective *in vitro* and *in vivo* against CD19dim cells, so that further research in this direction is necessary to be able to affirm that down-modulation of the target antigen is a mechanism involved in CD19-negative relapse after CAR T therapy.

### 2.6 Trogocytosis

The term trogocytosis is derived from the Greek “trogo”, meaning gnaw, and involves the transfer of plasma membrane proteins between two cells in the context of cell-cell contact.

Several reports have shown that lymphocytes can extract surface human leukocyte antigen (HLA) molecules from the antigen-presenting cells to which they are conjugated through the immunological synapse. This phenomenon was first identified by Hudrisier et al. (51) and has been documented in T, B and NK cells both *in vitro* and *in vivo*, constituting an important mechanism of immune function regulation and being possibly involved in the control of other cellular systems as well (52).

In CAR T cells, trogocytosis has been described by Hamieh et al. (53) who demonstrated, both in co-cultures and in an animal model, a transfer of the target antigen CD19 to CAR T cells from tumor cells engaged but not killed, leading to ineffective tumor lysis due to low target antigen density. This CAR T-driven trogocytosis invariably occurred in several cell lines and in primary CD19 leukemia blasts and was reversible, unlike other mechanisms that cause total loss of CD19 antigen in leukemic cells. In parallel, CD19 antigen transferred by trogocytosis transformed CD19+ CAR T cells into targets of neighboring CAR T cells that had not acquired the antigen, causing fratricidal killing.

## 3 Clinical data on CD19-negative relapse

### 3.1 CD19-negative relapse in tisagenlecleucel clinical trials

In 2013, Grupp et al. (4) reported the use of CTL019 produced at UPENN in two children with R/R ALL in the Children´s Hospital of Philadelphia (CHOP). Both children achieved CR of leukemia and robust expansion of CTL019 *in vivo*. However, two months after CTL019 infusion, patient 2 had a clinical relapse with CD19-negative blasts. This is the first CD19-negative relapse associated with CTL019 use reported in the literature. The emerging blasts were related to the initial clone, which share the same IGH sequence, consistent with potent antileukemic selective pressure fromCTL019 (4). In 2014, Maude et al. reported the use of CLT019 in 30 patients (25 children and five adults). One month after the infusion of CTL019, 27 patients were in CR. Seven patients subsequently had a relapse between six weeks and 8.5 months after infusion (4 CD19-positive and 3 CD19-negative relapses). Three out of four CD19-positive relapses were related to early loss of CTL019 in contrast with the three patients with CD19-negative relapse, in whichCTL019 cells were persistent at the time of relapse. There were no data to determine the mechanism involved in CD19-negative relapses, but one patient had received previous treatment with blinatumomab which may be related (9).

The FDA approval of tisagenlecleucel was based on the results of the ELIANA trial (NCT02435849) which was a one-cohort, open-label, multi-center, phase II study to determine the efficacy and safety of CTL019 in pediatric patients with R/R B-ALL. 92 patients were enrolled and 75 were infused. The overall remission rate within three months was 81%. At six months, the EFS and OS rates were 73% and 90%, respectively. Among patients with CR, 20 patients relapsed and two were classified as not responding to treatment because remission was not maintained for at least 28 days. Characterization of CD19 status revealed one patient having CD19-positive recurrence, 15 CD19-negative relapses and six with CD19 relapse unknown (5).

In the phase IIb expanded-access study focusing on pre-infusion exposure to blinatumomab and inotuzumab ozogamin (ELIANA confirmatory trial), 67 pediatric and young adult patients received tisagenlecleucel (15 with prior exposure to blinatumomab and nine to inotuzumab). The overall remission rate within three months was 85%. At 12 months, the OS rate was 83%. In total, 14 patients relapsed: nine (64%) CD19-negative relapses (two after blinatumomab) and five (35%) CD19-positive relapses (three after inotuzumab) without statistically significant associations due to the small number of patients (54).

### 3.2 CD19-negative relapse after tisagenlecleucel in real-world evidence

Tisagenlecleucel was approved for patients ≤25 years of age with second or subsequent R/R CD19+ B-ALL (U.S. and Europe) and any relapse after hematopoietic stem cell transplantation (Europe only) by the FDA in 2017 and the EMA in 2018. Since then, the real-world evidence from cohorts of patients treated with commercial Kymriah has increased rapidly.

The first experience in a real-world setting (outside clinical trials) was reported by Pasquini et al. of patients registered by the Center for International Blood and Marrow Transplant Research (CIBMTR). This non-interventional prospective study included patients who received tisagenlecleucel for an approved indication (pediatric/young adult patients with R/R ALL and adult non-Hodgkin’s lymphoma (NHL) patients). Among 255 B-ALL patients, the CR rate was 85.5%, with event-free survival (EFS) and overall survival (OS) of 52.4% and 77.2%, respectively, data that are comparable to those in the pivotal ELIANA trial. Unfortunately, one of the limitations of this registry is the lack of information related to CD19 status at relapse (12).

In May 2021, updating the French experience, Dourthe et al. reported on 51 patients with R/R B-ALL infused with tisagenlecleucel. The initial CR rate at day 28 was 96%. At the median follow-up (15.5 months), EFS and OS were 44% and 74%, respectively. Twenty-two of the 49 patients in CR relapsed with a median time of 3.7 months after CR. Twelve patients (55%) had a CD19-positive relapse and eight (36%) a CD19-negative relapse after a median time of 5.8 months (0–15) and three (1–7) months, respectively. Two patients had an unknown relapse. CD19-positive relapse was associated with loss of B-cell aplasia (BCA) and low tumor burden prior to lymphodepletion, while CD19-negative relapse was associated with high tumor burden and the use of corticosteroids. It should be noted that five of the eight CD19-negative relapse patients were pre-exposed to blinatumomab, although, due to the small number of patients, statistical significance was not reached (13).

The Pediatric Real World CAR Consortium (PRWCC) has reported the results of 185 children and young adults treated with tisagenlecleucel in a real-world setting (14). One hundred and sixty-one patients (85%) had CR at day +28, of whom 52 (37%) experienced disease relapse; among relapses, 59% (30 out of 52) were CD19-positive and 41% (22 out of 52) CD19-negative (three associated with myeloid transformation). In a second study of the same population included in the PRWCC, the authors studied the baseline characteristics of these patients in relation to its ability to predict relapse, as well as the outcomes of relapsing patients after treatment with tisagenlecleucel (15). Univariate analysis identified the number of pre-CAR relapses and post-CAR CD19-negative relapses (hazard ratio [HR]=1,59). Preinfusion high-disease burden, relapsed *vs* refractory status, and prior CD19-targeted therapy had high HRs for CD19-negative relapse risk but did not reach statistical significance.

Regarding the outcomes of relapsed patients, CD19-negative post-tisagenlecleucel relapse is associated with significantly decreased OS rates compared to CD19-positive relapse (12-month OS rate 30% *vs* 68%), which emphasizes the seriousness of this problem and the need to design strategies to prevent CD19-negative relapses.

The PRWCC collected retrospective data on infants (defined as children under 12 months of age at the time of the original diagnosis) with B-ALL who received commercial tisagenlecleucel. Of 14 infused patients, 12 (86%) had mixed lineage ALL with KMT2Ar. Nine patients (64%) achieved MRD-negative CR, with two experiencing relapse of the disease, one CD19-positive and the other CD19-negative with myeloid phenotype (16). The same PRWCC has reported on 55 patients with extramedullary (EM) disease before CAR therapy: central nervous system (CNS) EM, n= 40 and non-CNS EM, n = 15). All patients with EM disease were comparatively analyzed against the remaining patients in the PRWCC with bone marrow (BM)-only disease (n =129). Thirty-eight percent (15 out of 40) patients with CNS disease relapsed post-tisagenlecleucel, five of whom experienced a CD19-negative relapse. Forty percent (six out of 15) of patients with non-CNS EMd isease relapsed post-tisagenlecleucel, one of whom experienced a CD19-negative relapse.CD19-negative relapse was significantly more frequent in BM-only disease (20 out of 38 patients) *vs* CNS (five out of 15) and non-CNS EM (one out of six) (55).

### 3.3 CD19-negative relapse in clinical trials with anti-CD19 CART cells other than tisagenlecleucel

Other CAR T cells with 4-1BB or CD28 costimulatory domains have been tested in R/R B ALL patients (both children and adults) in several clinical trials.

The Seattle research group designed a CART product with a defined 1:1 CD 4+:CD8+ CAR-T cell ratio. The CAR construct comprised an FMC63-derived CD19-specific scFv, a 4-1BB costimulatory domain and a CD3ζ signaling domain. In the PLAT-02 phase I study, 45 children and young adults were enrolled and 43 were infused. The majority (88.37%) achieved MRD-negative CR and 13 underwent allogeneic hematopoietic stem cell transplant (alloHSCT). Of these, ten remained in remission but three subsequently relapsed (two positive-CD19 relapses and one negative-CD19 relapse). Among 25 subjects that did not undergo alloHSCT, 20 relapsed, 10 with CD19-negative disease and 10 with CD19-positivedisease. Shorter duration of BCA was a risk factor for CD19-positive relapse, but no significant risk factors were found for CD19-negative relapse (56).

At the Hospital Clinic in Barcelona (Spain), an academic CD19-41BB CAR-T cell (AR-0001) was developed. In the CART19-BE-01 trial, 47 patients received ARI-0001, 38 with R/R B-ALL (11 children). MRD-negative CR was reached in 84.2%of these patients, with PFS and OS rates at 1 year of 47% (27%–67%) and 68.6% (49%–88%) for the whole ALL cohort, respectively. Fifteen patients relapsed after ARI-0001 cell infusion. Most relapses (87%) were CD19-positive, while just two (13%) were CD19-negative (57). Again, a short persistence of CART cells could explain this phenomenon, but the low number of patients prevented the authors from identifying any relationship of another factor with CD19-negative relapse.

The most important clinical trial testing a CD28-costimulated anti-CD19 CAR T cell in ALL is ZUMA-3, whose Phase II has driven the FDA and EMA approval of KTE-X19 (brexucabtageneautoleucel, Tecartus^©^) for commercial use in adults with R/R B-ALL. In Phase 1 of ZUMA-3 (19), of the 45 patients treated, 53% achieved CR and 16% CR with incomplete hematological recovery (CRi). Of the 13 patients who relapsed, three (23%) had a CD19-negative relapse, and seven had a CD19-positive relapse, with no data available for three of the patients. In Phase II of ZUMA-3 (20), KTE-X19 was administered to 55 patients, with 39 patients (71%) achieving CR or CRi. At data cutoff, 12 (31%) relapsed; three out of nine patients, with available data at relapse (33%), had CD19-negative relapse.

Besides the ZUMA-3 trial, at least five additional clinical trials on R/R B-ALL patients reported data regarding CD19 status at relapse after CD19-28 CAR T cell therapy (**Table 1**).

**Table 1:** Clinical outcomes after CD19 CAR T cell therapy in patients with R/R B-ALL.

Study (reference)	N Population (infused)	CAR design	Vector	CAR T cell dose (cells/kg)	Response, CR, (%)	Relapserate (%)^*^	CD 19 negativerelapserate (%)	Mechanism
TisagenlecleucelClinical Trials								
Phase I								
Grupp et al. (4)	2 (2) children and AYA	CTL019	lentiviral	1.4×10^6^ to 1.2×10^7^	CR: 100 (2/2)	100 (2/2)	100 (2/2)	N/A
Maude et al. (9)	30 (30) children and AYA	CTL019	lentiviral	0.2×10^6^ to 5.4×10^6^	CR: 90 (27/30) at 1 mo after infusion	25,9 (7/27) 6 we and 8.5 mo after infusion	42,8 (3/7)	1 prior blinatumumab
Phase II								
Maude et al EIANA (5)	92 (75) children	CTL019	lentiviral	2.0-5.0 x 10^6^ (<50 kg) 1.0-2.5 x 10^8^(>50 kg)	CR: 81(61/75) at 3 mo after infusion OS and EFS: 73 and 98, at 6 mo after infusion	32,7 (22/61)	68.18 (15/22) (unknown 27,27)	N/A
Phase IIb								
Si Lim et al. ELIANA (54)	67 (67) children and AYA	CTL019	lentiviral	2.0-5.0 x 10^6^ (<50 kg) 1.0-2.5 x 10^8^(>50 kg)	CR 85 (57/67) at 3 mo after infusion	20,9 (14/57)	64 (9/14)	2 (22,2%) prior blinatumomab
Tisagenlecleucel outside clinical trials. Real-World Evidence.								
Pasquini et al. (12)	255 (255) children and AYA	CTL019	lentiviral	2.0-5.0 x 10^6^ (<50 kg) 1.0-2.5 x 10^8^(>50 kg) commercial	CR 85.5 (218/255) OS and EFS: 77.2 and 52.4	47.6 (121/255)	N/A	N/A
Dourthe et al. (13)	51 (51)children and AYA	CTL019	lentiviral	commercial	CR 96 (49/51) at 28 days after infusion OS and EFS: 74 and 44	44.8 (22/49)	36 (8/22)	Associated to high tumor burden and use of corticosteroids 5 prior blinatumomab
Schultz et al. (15)	182 (182) children and AYA	CTL019	lentiviral	commercial	CR 88.5 (161/182)	37 (52/162)	41 (22/52)	3 associated a myeloid transformation
Moskop et al. (16)	14 children	CTL019	lentiviral	commercial	CR 64 (9/14)	22.2 (2/9)	50 (1/2)	Myeloid transformation
Fabrizio et al. (55)	55 children with extramedullary disease	CTL019	lentiviral	comercial	Not evaluable	38 (15/40) CNS 40 (6/15) no CNS	33.3 (5/15) CNS 16.7 (1/6) no	N/A
Clinical trials anti CD19 CAR-T cells other than tisagenlecleucel								
Finney et al. (56)	45 (43) children and AYA	FMC63–CD28-4-1BB	lentivirus	2 x 10^5^ CD4+:CD8+=1	CR: 88,37 (38/43)	53,5(23/43)	47,8 (11/23)	N/A Not risk factor founded
Ortiz-Maldonado et al. (57)	58 in total (38 R/R B-ALL)	ARI-0001	lentivirus	0,4-5 x 10^5^	CR 84.2 (35/38) OS and EFS at 1 ye 68.6 and 47	42,8 (15/35)	13,3 (2/15)	N/A
Shah NN et al. (58)	50 (50) (children and AYA)	FMC63-CD28	γretrovirus	Two dose levels: DL 1: 1 × 10^6^; DL2 3 × 10^6^	CR: 62 (31/50) OS and EFS: 10.5 and 3.1 mo	18 (7/31)	42,8 (3/7) (14,28 (1/7) unknown)	N/A
Lee et al. (8)	20 (20) children and AYA	FMC63–CD28	γretrovirus	0,03-3 x 10^6^; dose scalation	CR: 70 (14/20) OS 51 at 10 mo	14,3 (2/14)	100 (2/2)	N/A
Gardner RA et al. (7)	45 (43) children and AYA	FMC63-CD28	lentivirus	1 x 10^6^ CD4+:CD8+=1	CR 93 (40/43) OS and EFS at 1 ye 50.8 and 69.5	45 (18/40)	38,8 (7/18)	1 linage switch
Park et al. (10)	83 (53) children and AYA	SJ25-CD28	γretrovirus	1.5-3 x 10^6^	CR: 83 (44/53) OS and EFS 20.1 and 10.6 mo	56,81 (25/44)	16 (4/21)	N/A
Jacoby et al. (59)	21 (20) children and AYA	FMC63–CD28	γretrovirus	1 x 10^6^	CR 90 (18/20)	22,2 (4/18)	75 (3/4) 25 (1/4) unknown	N/A Not risk factor founded
Zhang et al. (60)	115 (110) Children and adults	FMC63–CD28 or 4-1BB	lentivirus	1-10 x 10^5^	CR 93 (102/110) OS and EFS at 1 ye 58 and 64	22,5 (23/102)	30 (7/23)	N/A Not relation founded between CAR domains

^*^calculated on the responding population.

R/R B-ALL, relapse/refractory B acute lymphoblastic leukemia; CR, complete remission; OS, overall survival; EFS, event free survival; mo, months; ye, years; da, day; N/A, not available; CNS, Central nervous system; AYA, adolescents and young adults.

Data included CD19 relapse rate and its mechanism if it was described.

1) A phase 1 trial evaluated an anti-CD19-28 CAR in 21 young patients (20 with R/R B-ALL and one with LNH, aged 1-30 years). Of the 20 patients with B-ALL, the CR rate was 70%, with 12 out of 20 achieving MRD-negative CR. The response of ten patients was consolidated with alloHSCT who remained disease-free unlike the other two patients with MRD-negative disease who were not eligible for alloHSCT and relapsed with CD19-negative disease at three and five months, respectively (8).

2) Another phase 1 clinical trial with a CD19 CAR product of defined CD4/CD8 composition with limited effector differentiation was developed. Forty-five children and young adults (aged 1.3-25.3 years) were enrolled and 43 were infused. The rate of MRD-negative CR was 93% and the median follow-up was 9.6 months. Eighteen of the 40 MRD-negative CR patients (45%) relapsed, seven with undetectable CD19+ cells (39% of relapses) including one lineage switch to AML occurring one month after CAR T infusion. A risk factor for relapse with CD19-positive disease was a short duration of BCA which may be favored by low quantities of CD19+ B cells (malignant and non-malignant) at the time of infusion. No risk factors were identified for CD19-negative relapse (7).

3) Park et al. published the long-term results of another phase I trial that enrolled 83 adults, with 53 of them receiving CAR Infusion. Of the 53 patients, forty-four (83%) reached CR, and thirty-two had MRD-negative CR. The median follow-up for EFS and OS was 29, 10.6 and 20.1 months, respectively. All nine patients who had MRD-positive CR after CAR-T relapsed with CD19-positive disease, while 16 of the 32 patients with MRD-negative CR relapsed (four with CD19 negative disease). In this cohort, CD19-negative relapse was just 16% (10).

4) The Sheba Medical Center in Israel developed a phase Ib/II study where 21 patients (median age 11 years, range 5-48) were enrolled and 20 infused with an autologous FMC63 CART-derived cell with a CD28 costimulatory domain. The remission rate was 90%. Four responding patients relapsed, three with CD19-positive relapse and one with unknown CD19 status at relapse (59).

5) Finally, in a long-term report (median follow-up 4.8 years) on 50 infused children and young adults, with a CAR developed at the National Cancer Institute,31(62%) achieved CR, with 28patients being MRD-negative. Of these patients, 21proceeded to consolidative alloHSCT. Two patients after alloHSCT and the seven MRD-negative patients who did not receive alloHSCT relapsed, three with CD19-positive relapse, three with CD19-negative/dim disease, and one with unknown CD19 status at a median of 152 days post-CAR infusion (58).

Prospective randomized trials comparing the results of anti-CD19 CAR T cells co-stimulated with 41BB or CD28 are lacking, so it is currently not possible to determine whether either offers advantages in terms of efficacy in R/R ALL. A study comparing these two types of CAR T cells in ALL in a murine model was carried out, with 36 patients also studied retrospectively (61). This study concludes that CD19-41BB CAR T cells have advantages in terms of persistence both in the murine model and in the patients studied retrospectively, which would confer greater antitumor efficacy in EFS and risk of CD19-positive relapses, but logically, it is not possible to extrapolate any data regarding potential efficacy against CD19-negative relapses. These 36 patients were part of a phase I/II trial in which a total of 110 R/R ALL patients were infused, 21 with a CAR CD19-28 product and 89 with CD19- 41BB. Among 102 evaluable patients,23 (22.5%) relapsed, seven of them (30%) with CD19-negative disease (60).The authors found no significant differences between the costimulatory domains of the CAR products received by relapsed patients or any other factors related to CD19-negative relapse.

A meta-analysis published in 2021 included 15 clinical trials with 41BB-and CD28-costimulated second-generation CAR T cell therapies (11 and three studies, respectively), as well as a fourth-generation anti-CD19 CAR T cell therapy (one study). Eleven of the 15 trials presented data on the incidence of relapse and their CD19-positive or CD19-negative phenotype (62). Of the 82 relapse cases, CD19 status was unknown in ten cases, 27 had CD19-positive disease, 42 had CD19-negative disease, and three experienced a lineage switch to AML. The cumulative incidence of CD19-positive or negative recurrences in each treatment subgroup (CAR T CAD19-41BB, CD19-CD28 and fourth-generation CAR T cell therapy) was not reported, but the overall cumulative incidence of recurrences was not significantly different among the three treatment subgroups (62).

Reading through all the available data on post-CD19 CAR relapse in B-ALL patients in clinical trials and real-world evidence, CD19-negative relapse accounts for a non-negligible percentage of cases. However, perhaps due to the small number of patients, the different studies have not been able to establish risk factors as clearly as in CD19-positive relapse, in which the post-infusion behavior of CAR T cells seems to be highly predictive.

To address the potential pre-infusion characteristics that constitute risk factors for relapse and the relapse phenotype, two retrospective multicenter studies were conducted on 420 children and young adults who received CD19CAR treatment, either commercially available tisagenlecleucel or another CD19-CAR T cell therapy, in six clinical trials (NCT01626495, NCT02906371, NCT02028455, NCT02625480, NCT02435849, and NCT01593696) for R/R B-ALL across seven centers in the USA.CAR constructs included two CD194-1BB constructs (tisagenlecleucel and SCRI-CAR19) and one CD19-CD28 construct (17, 18).The first study focused on the impact of prior blinatumomab exposure on subsequent CD19CAR outcomes and demonstrated that blinatumomab non-responders had lower CR rates and a higher cumulative incidence of relapse than either blinatumomab responders or blinatumomab-naïve patients (17). A second study of this dataset aimed specifically to characterize pre-infusion risk factors associated with each pattern of relapse, that was either positive or negative for CD19 expression (18). Of 420CART treated patients, 166 (39.5%) relapsed, including 83 (50%) CD19-positive patients, 68 (41%) CD19-negative patients, and 12 (7.2%) patients with lineage-switched relapses. Two or more previous remissions was the only variable independently associated with an increased risk of CD19-positive relapse, while*KMT2A* rearrangement was the only pre-infusion risk factor associated with lineage switching. CD19-negative relapses were associated with children under the age of seven at CD19-CAR infusion, 4-1BB CAR type, prior blinatumomab non-response, and a high disease burden (>5% blasts) pre-CAR (18).

## 4 Strategies to overcome CD19-negative relapse

Relapse of ALL post-CAR, whether CD19-positive or CD19-negative, is a very important problem which constitutes the main limitation of an otherwise very successful therapy in patients with R/R ALL. The prognosis of patients who relapse after CAR T treatment is poor, particularly in cases of CD19-negative relapse. For this reason, the question has been raised as to whether early allogeneic transplantation should be performed early on as a consolidation therapy in all cases of CR post-CAR T cell therapy or whether it should at least be performed in patients who meet any of the factors identified as high-risk criteria for relapse (13, 63). The analysis of this issue is beyond the scope of this paper, but there are very interesting recent reviews on the subject, which are highly recommended (64–67).

**Figure 2:**
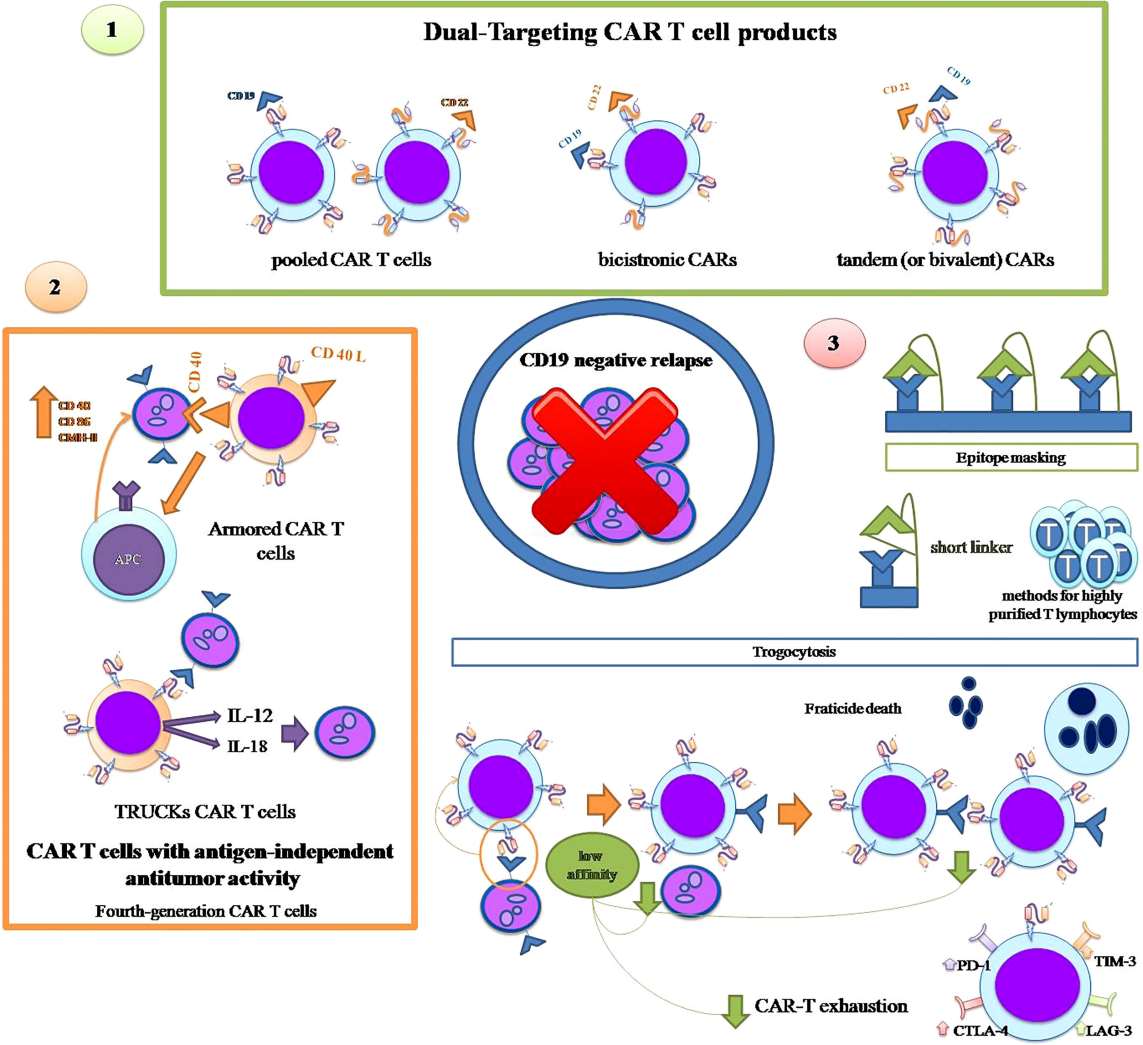
Strategies to overcome CD19-negative relapse: 1. Dual Targeting CAR-T cell products: use of pooled CAR-T cells with different unique specificities, bicistronic CARs and tandem CARs. 2. CAR-T cells with antigen-independent antitumor activity: armored CAR T cells and TRUCKs CART cells. 3. Specific strategies against certain mechanisms: epitope masking and trogocytosis.

Here we discuss specific strategies (represented in **Figure 2**), other than HSCT as consolidation, that have been developed at both the clinical and CART cell design levels, to try to prevent CD19-negative relapse of leukemia.

### 4.1 Dual-targeting CAR T cell products

The most intuitive alternative strategy to prevent leukemic relapse due to CD19-negative cells is the use of CAR T cells with more than one antigenic specificity, either by combining several CAR T cells with different unique specificities (pooled CAR T cells) or by using a single CAR T product with more than one specificity i.e. at least one antigenic specificity in addition to CD19. The latter can, in turn, be carried out with two types of products: tandem (or bivalent) CARs that contain two antigenic specificities in a single extracellular domain, and bicistronic CARs that are transduced using a single vector encoding two distinct CARs that are expressed in two separate extracellular receptors (68, 69).

CD22 is an adhesion molecule almost exclusively restricted to the B cell lineage and expressed in most B-ALL cases (70–73), making CD22 an ideal candidate as a second antigenic specificity added to CD19 for the dual approach to CAR T therapy in ALL (74). In addition, CD22 is preserved in many cases of CD19-negative relapse after CD19 CAR T treatment.

In a clinical trial, 15 B-ALL children and adults were treated with an anti-CD22 CAR T cell therapy after anti-CD19 CAR T treatment, with nine cases of CD19 dim/negative relapse; five out of five patients with CD19 dim/negative relapse, who received the 1x10^6^ CD22 CAR T cell dosage, obtained CR which demonstrates the potential usefulness of anti-CD22 CAR T cell therapy for these patients (49). However, in the vast majority of cases, relapses after CR with the anti-CD22 CAR product were related to the decrease in CD22 expression due to reduced antigen density, which has been observed in other anti-CD22 CAR T trials (75–77). This makes the sequential administration of CAR T therapy targeting CD19 and CD22 questionable and has prompted several strategies for both sequential infusion with only 1-2 day intervals and cocktail co-administration, as well as new bicistronic and tandem CAR T products. **Table 2** summarizes the published clinical trials with the anti-CD19 CAR T product plus the anti-CD22 approach for B-ALL patients (75–84).

**Table 2:** Clinical outcomes after CD19 and CD 22 CAR T cell therapy in patients with R/R B-ALL.

Sequential or Cocktail CD19 + CD22 infusion									
Study (reference)	N Population (infused)	CD 19 CAR T cell dose (cells/kg)	CD22 infusion	CD 22 CAR T cell dose (cells/kg)	CR after CD 19 infusion %	CR after CD 22 infusion %	Relapserate %	Relapse CD 19 negativerate %	Descriptionrelapse
Pan et al. (78)	20 children (20)	Median 10 (3.3-42.8) x 10^5^	CD19 CAR T cells became undetectable by FCM in peripheral blood	Median: 10 (0.25-47.4) x 10^5^	100 (20/20)	100 (20/20)	15 (3/20)	66.6 (2/3)	1 CD19^+^/CD22^dim^ 2 CD19^-^/CD22^N/A^
Liu Am et al. (76)	27 children and AYA (27 CD19 and 21 CD19/CD22)	Median: 1.0 (0.486–5.0) × 10^5^	Based in clinical condition (at least 1 month within 6 months after the CD19 CAR infusion)	Median: 2.0 (0.32–5.0) × 10^5^.	85 (23/27)	76.2 (16/21)	33.33(7/21)	28.6 (2/7)	2 CD 19 ^-^ 4 CD19^+^ 1 N/A BCR-ABL CD22^N/A^
Zhang et al. (77)	4 adults (4)	1 × 10^6^	Day 1 CD22 infusion Day 2 CD19 infusion	1 × 10^6^	100 (4/4)		75 (3/4)	66.7 (2/3)	1 CD19 ^+^ 2 CD19 ^-^/CD22^dim^
Wang et al. (79)	105 R/R B-ALL/NHL (51 R/R B-ALL)	2.6 ± 1.5 x 10^6^	infused separately on successive days	2.7 ± 1.2 X 10^6^	94.11 (48/51)		49 (24/49)	4 (1/24)	23 CD19^+^/CD22^+^ 1 CD19^-^/CD22^dim^
Yan et al. (80)	23 adults (23)	2.1 (1 -4.2) x 10^6^	infused separately on successive days	2 (0.9- 4.88) x 10^6^	95.7 (22/23)		30.4 (7/23)	28.6 (2/7)	5 CD19^+^/CD22^+^ 2 CD19 ^-^/CD22^dim^
Coadministration									
Study (reference)	N Population (infused)	Ratio	CAR T cell dose (range) (cells/kg)	CD 22 CAR T cell dose (cells/kg)	CR after CD 19 infusion %	CR after CD 22 infusion (%)	Relapserate (%)	Relapse CD 19 negativerate (%)	Descriptionrelapse
Whang et al. (81)	232 children and AYA (225)	1:1 (median 0.94)	5.6 (1.3- 13.0) x 10^6^	2.8 (2.1-4.0) x 10^6^	2.7 (1.9-3.7) x 10^6^	96.9 (192/194 evaluable patients)	22.2 (43/194)	39.5 (17/43)	24 CD19^+^/CD22^+^ 16 CD19^-^/CD22^dim^ 1 CD19 ^-^/CD22^-^ 2 unknown
Tandem									
Study (reference)	N Population (infused)	Design	CAR T cell dose (cells/kg)		CR after infusion (%)		Relapserate (%)	Relapse CD 19 negativerate (%)	Descriptionrelapse
Spiegel et al. (82)	17 adults (17)	CD19-22.BB.z-CAR	Doseescalation: DL1: 1 × 10^6^ DL2: 3 × 10^6^ DL3: 1 × 10^7^		82.3 (14/17)		64.3 (9/14)	44.4 (4/9)	4 CD19^+^/CD22^+^ 1 CD19^+^/CD22^N/A^ 3 CD19 ^-^/CD22^dim^ 1 CD19^low^/CD22^+^
Dai et al. (75)	6 adult (6)	CD19- CD22-BB-z	1.7 to 3 × 10^6^		100 (6/6)		50 (3/6)	33.3 (1/3)	2 CD19^+^/CD22^+^ 1 CD19-/CD22 ^low^
Bicistronic									
Study (reference)	N Population (infused)	Vector	CAR T cell dose (cells/kg)		CR after infusion (%)		Relapserate (%)	Relapse CD 19 negativerate (%)	Descriptionrelapse
Cordoba et al. (83)	23 children and AYA (23)	CD19.OX40/22.BBz	Doseescalation: DL1: 0.3–2 × 10^6^ DL2: 3 × 10^6^ DL3: 4.3–5 × 10^6^		86.6 (13/15)		69.2 (9/13)	33.3 (3/9)	1 CD19-/CD22 ^N/A^ 1 CD19-/CD22 ^low^ 1 CD19-/CD22 ^–^ 6 N/A
Shalabi et al. (84)	20 children and AYA (15)	CD19.28z/22.BBz	Doseescalation: DL1 3x 10^5^, DL2: 1 x 10^6^ DL3: 3x 10^6^.		60 (12/20)		25 (4/12)	25 (1/4)	3 CD19^+^ 1 CD19^-^ CD 22^N/A^

R/R B-ALL, relapse/refractory B acute lymphoblastic leukemia; CR, complete remission; N/A, not available; NHL, Non-Hodgkin lymphoma; FMC, flow cytometry; DL, Dose level.

None of the published clinical trials summarized in **Table 2**, including different CD19/CD22 dual-targeting strategies, have completely eliminated the occurrence of CD19-negative relapses, so research in this area continues actively, with the design of new and potentially more active CD19/CD22 CAR constructs (85), as well as the inclusion of a second antigenic specificity in addition to CD19 other than CD22. Ruella et al. found that the IL-3 receptor αchain (CD123) was highly expressed on leukemia-initiating cells and on CD19-negative blasts at relapse after CAR-T19 administration and determined that CART123 eradicated CD19-negative leukemia. In addition, the combination of CART19 and CART123 prevented antigen-loss relapse in xenograft models, while the CD19/CD123 bicistronic CAR-T cell product had superior *in vivo* activity against B-ALL as compared to each single-expressing CAR-T or pooled combination CAR-T (86). Another second potential target is the B cell-activating factor receptor (BAFF-R). *In vitro*, CD19/BAFF-R dual CAR-T cells exhibited cytotoxicity against both CD19−/− and BAFF-R−/− human ALL cells; and *in vivo*, a single dose of CD19/BAFF-R dual CAR-T cells eradicated both CD19−/− and BAFF-R−/− ALL variants, whereas monospecific CD19 or BAFF-R CAR-T cells allowed outgrowths of CD19−/BAFF-R+ or CD19+/BAFF-R− tumors, respectively (87). CD20 has also been investigated as a potential target in combination with CD19 and CD22 in a tricistronic CAR construct (88); anti-CD19/CD20/CD22 CART-cells lysed CD19-negative blasts from patients who relapsed after CD19CAR T cell therapy both *in vitro* and in an animal model and were as effective as CD19 CAR-T cells against primary CD19-positive disease.

### 4.2 CAR T cells with antigen-independent antitumor activity

Along with these strategies giving CAR-T cells more than one antigenic specificity, constructs have also been developed that provide CAR-T cells with tools to lyse tumor cells independently of target antigen expression so that the antitumor effect can be maintained even in the event of antigen loss.

Fourth-generation CAR-T cells, also known as armored CAR-T cells, are engineered to express proteins alongside a second- or third-generation CAR to enhance the efficacy of CAR-T cells, reduce the immunosuppressive milieu of the tumor microenvironment and to induce natural anti-tumor T cells that act in an antigen-independent manner.

The armored CAR-T cells that constitutively express CD40L can engage CD40-positive tumor cells and kill them directly, while activating neighboring antigen presenting cells (APCs) that increase the tumor expression of co-stimulatory molecules such as CD40, CD86 and the class II major histocompatibility complex (MHC), enabling lysis of tumor cells by endogenous T cells even in the absence of target antigen (89).

T-cells redirected for universal cytokine killing (TRUCKs) are another type of fourth-generation CAR T cells that release proteins after engagement with the target antigen. TRUCKs have been designed to secrete IL12 (90, 91) or IL18 (92) that attract and activate endogenous immune cells to eliminate antigen-negative tumor cells.

### 4.3 Strategies for specific mechanisms of antigen loss

Besides these general strategies of multitargeting or target independent lysis, some other approaches have been designed for specific mechanisms of CD19 antigen loss after CD19 CAR T therapy.

There are at least two proposed strategies to overcome epitope masking due to accidental transduction of blasts with the anti-CD19 CAR construct during the manufacturing process. A new design of the CAR construct that includes a short linker prevents its cis-binding to CD19 on the surface of blasts and thus epitope masking, allowing anti-CD19 CAR-T cells to recognize and attack CAR-transduced blasts (93). Ruella et al., who originally described the phenomenon of epitope masking, have developed an anti-CD19 CAR idiotype that can recognize and eliminate transduced B-ALL blasts (94). Although these solutions have undeniable potential, the most important strategy to prevent accidental transduction of ALL cells and epitope masking is the application of methods for highly purified T lymphocytes in the apheresis product to avoid the presence of blasts in the starting material that could be accidentally transduced.

Regarding trogocytosis, there is currently no fully defined strategy to avoid this phenomenon and its two deleterious consequences, the loss of CD19 antigen by leukemic cells and fratricide. However, very recently, it has been demonstrated, both *in vitro* and in an animal model, that low affinity anti-CD19 CAR-T cells show reduced trogocytosis which prevents antigen loss in leukemic cells, while maintaining a lytic enzyme and interferon production capacity identical to CAR-T cells with the high-affinity CD19 binding domain FMC63 (95). In addition to the reduction of trogocytosis, other advantages have been invoked for low-affinity CAR-T cells, mainly lower toxicity and greater persistence due to the absence of cellular exhaustion, while exhibiting enhanced proliferative and *in vivo* antitumor activity, even in low-antigen density cell lines, as compared to FMC63 CAR T cells (96). However, two ALL clinical trials, one on pediatric patients and the other on adults with the same low-affinity CAR-T cell product (named CAT), have not prevented CD-19-negative relapses from occurring in five out of 12 children and two out of 20 adults, respectively (96, 97). These two studies do not provide information on the mechanism of antigen loss in CD19-negative relapsing patients, so it is not possible to know whether trogocytosis was responsible for the CD19-negative relapses in these low-affinity CAR-T-treated patients.

Finally, as mentioned above, it is not entirely clear that antigen down-modulation acts as the sole causal mechanism of CD19-negative relapse, but CD19 dim relapses occur in some ALL patients. Unlike native TCR, CD19 CAR antitumor activity is antigen density-dependent and fails when the number of surface antigen molecules falls below a certain threshold, resulting in the escape of antigen-low cellular variants that lead to resistance to therapy. Increasing the signal strength allows recognition of cells with low antigenic density, which can be achieved through the substitution of a CAR’s CD8 hinge-transmembrane (H/T) region with a CD28H/T, as well as through the inclusion of additional immunoreceptor tyrosine-based activation motifs (ITAMs) (46). Another strategy, with its antitumor effect on cells with low antigenic density, is TRAC locus editing which establishes in T cells a new antigen receptor that incorporates into the TCR–CD3 complex the same heavy and light chains as those assembled into a scFv gene in a corresponding CAR. These receptors, termed HLA-independent TCRs or HIT receptors, are more sensitive than CARs and offer new perspectives for the treatment of ALL variants with low antigen density (98).

## 5 Conclusion

Anti-CD19 CAR-T therapy has brought about a substantial change in the prognosis for patients with R/R ALL, for whom therapeutic options were very limited. Overall, about 50% of treated patients achieve prolonged remission, but the remaining cases relapse. CD19-negative relapse accounts for between 20 and 40% of cases (**Table 1**) and invariably carry a worse prognosis because the possibilities of a new treatment based on immunotherapy are excluded. Multiple mechanisms that affect CD19-negative relapse have been described including genetic mutations, alternative splicing, epitope masking, decreased CD19 antigen density, trogocytosis and lineage switch, although the actual clinical incidence of each of these mechanisms is not completely known. Strategies to avoid CD19-negative relapses regardless of the mechanism that causes them include the use of CAR-T cell therapy with more than one antigenic specificity, generally CD19 and CD22, and fourth-generation CAR-T cells. Dual CD19/CD22 CAR T cells have not completely prevented CD19-negative recurrences, since some cases have occurred in all the published series, while, with regard to fourth-generation CAR-T cells, there are insufficient clinical data to draw conclusions. Likewise, the data we have on specific strategies for each of the CD19-negative relapse mechanisms are very limited. Research in this field is very active and is essential to solve the most important problem with an otherwise life-saving therapy for this group of patients.

## Author contributions

CA-P and CH: Study conception and design. CA-P, MDC and CH: Searching and selecting publications. CA-P and MDC: Writing original draft preparation. CH: writing, review and editing. KB and CH: supervision. All authors contributed to the article and approved the submitted version.

## Glossary

**Table:**

AML	Acute Myeloid Leukemia
APCs	antigen presenting cells
AS	Alternative splicing
B-ALL	B cell acute lymphoblastis leukemia
BAFF-R	B cell activating factor receptor
BCA	B cell aplasia
CAR	Chimeric antigen receptor
CAT	low affinity CAR
CD	Cluster of differentation
CHOP	Children´s Hospital of Philadelphia
CIBMTR	Center for International Blood and Marrow Transplant Research
CLL	Chronic lymphocytic leukemia
CNS	Central nervous system
CR	Complete remission
CRi	CR with incomplete hematological recovery
CRISPR	Clustered Regularly Interspaced Short Palindromic Repeats
CRS	Cytokine release syndrome
DNA	Desoxiribonucleic Acid
EFS	event-free survival
EM	extramedullary
EMA	European Medicines Agency
FDA	Food and Drug Administration
H/T	hinge-transmembrane
HLA	Human leukocyte antigen
HR	Hazard ratio
alloHSCT	Allogeneic hematopoietic stem cell transplant
IGH	Immunoglobulin heavy chain
ITAMs	Immunoreceptor tyrosine-based activation motifs
IL	Interleukin
MHC	major histocompatibility complex
MRD	Minimal residual disease
NHL	non-Hodgkin’s lymphoma
OS	overall survival
PRWCC	Pediatric Real World CAR Consortium
PFS	progression-free survival
R/R	refractory or relapsed
rMATS	Multivariate Analysis of Transcript Splicing
RNA	Ribonucleic Acid
mRNA	messenger RNA
scRNA-sep	single-cell RNA sequencing
scFvs	single-chain fragment variables
TCR	T cell receptor
TRAC	T-cell receptor α constant
TRUCKs	T-cells redirected for universal cytokine killing
UPENN	University of Pennsylvania
Δex2	Isoform of exon 2
